# Transgelin as a potential target in the reversibility of pulmonary arterial hypertension secondary to congenital heart disease

**DOI:** 10.1111/jcmm.13912

**Published:** 2018-10-18

**Authors:** Li Huang, Li Li, Tao Yang, Wen Li, Li Song, Xianmin Meng, Qing Gu, Changming Xiong, Jianguo He

**Affiliations:** ^1^ Center of Pulmonary Vascular Disease State Key Laboratory of Cardiovascular Disease Fuwai Hospital National Center for Cardiovascular Diseases Chinese Academy of Medical Sciences and Peking Union Medical College Beijing China; ^2^ Department of Pathology State Key Laboratory of Cardiovascular Disease Fuwai Hospital National Center for Cardiovascular Diseases Chinese Academy of Medical Sciences and Peking Union Medical College Beijing China; ^3^ State Key Laboratory of Cardiovascular Disease Fuwai Hospital National Center for Cardiovascular Diseases Chinese Academy of Medical Sciences and Peking Union Medical College Beijing China

**Keywords:** congenital heart disease, pulmonary arterial hypertension, reversibility, transgelin

## Abstract

**Background:**

The reversibility of pulmonary arterial hypertension (PAH) in congenital heart disease (CHD) is of great importance for the operability of CHD. Proteomics analysis found that transgelin was significantly up‐regulated in the lung tissue of CHD‐PAH patients, especially in the irreversible group. However, how exactly it participated in CHD‐PAH development is unknown.

**Methods:**

Immunohistochemical staining and Western blot were performed for further qualitative and quantitative analysis of transgelin in the lung tissues of CHD‐PAH patients. The mechanism of transgelin in CHD‐PAH development was explored in vitro. Primary human pulmonary arterial smooth muscle cells (hPASMCs) were cultured and infected with TAGLN siRNA or TAGLN lentiviral vector. Cell morphologic change (Coomassie Brilliant Blue staining), proliferation (cell count and EdU assay), apoptosis (terminal deoxyribonucleotidyl transferase mediated dUTP nick end labeling assay and Annexin‐V flow cytometry) and migration (transwell) were evaluated following the cell treatment. The mRNA and protein expression levels were detected in real‐time PCR and Western blot.

**Results:**

In line with the proteomic findings, transgelin was obviously expressed in PASMC of the middle pulmonary arterioles, especially in the irreversible PAH group. Also, transgelin expression showed positive relation with pathological grading. Experiment in vitro demonstrated that transgelin overexpression promoted PASMC proliferation and migration, strengthened cytoskeleton and was accompanied by increased expression of synthetic phenotype markers (osteopontin, proliferating cell nuclear antigen) and anti‐apoptotic protein (bcl‐2). On the other hand, suppression of transgelin expression activated PASMC apoptosis, reducing cell proliferation and migration.

**Conclusions:**

Transgelin may be a potential target in the development of irreversible CHD‐PAH through inducing PASMC phenotype change, proliferation, migration and reducing cell apoptosis.

## 1 INTRODUCTION

Pulmonary arterial hypertension (PAH), characterized by increased pulmonary arterial pressure (PAP), pulmonary vascular resistance (PVR) and aggravated right heart function, is a common combination of congenital heart disease (CHD). Long‐time exposure to high blood flow in CHD patients can lead to endothelial dysfunction and pulmonary vascular remodelling, which may finally result in irreversible pulmonary vasculopathy. Studies showed prevalence from 4% to 28%,^1^, ^2^ and 30% of unrepaired CHD patients have PAH.^3^ Our previous data showed that 47.5% of Chinese CHD patients complicated by PAH.^4^ Interventional and surgical repair as the main treatment for CHD has proved to be highly beneficial for the patients. But a study showed that 14% of patients were affected with PAH after the correction of complete atrioventricular septal defects, and the 1‐year mortality of CHD‐PAH patients who underwent cardiac operations reached 18.5%.^3^ Studies even showed a worse 10‐year post‐operative survival for patients with persistent post‐operative PAH than for those with Eisenmenger syndrome,^5^, ^6^ which was considered the severe and irreversible condition of CHD‐PAH. It means that the pulmonary vascular lesion had become irreversible and the abnormality in haemodynamics cannot be reversed by surgical repair in these patients. Furthermore, the condition may be even worse because of the sudden haemodynamics change after surgery. Despite advances in our understanding of the pathophysiology and the management of PAH, significant translational and therapeutic gaps remained for the medication.^7^ That raised the importance of accurate evaluation and gaining a better understanding about the reversibility of PAH in CHD patients before surgery. As of now little is known about that.

To explore the pathogenetic mechanism of irreversible CHD‐PAH, proteomics comparison of the lung tissues in both reversible and irreversible CHD‐PAH patients as well as in normal were performed. Proteomic analysis showed that transgelin was significantly up‐regulated (2.5‐fold) in irreversible CHD‐PAH group compared to the reversible and control group.^8^ Immunohistochemical staining and Western blot also confirmed the findings, and the expression of transgelin was significantly positively related with pathological grading of pulmonary arterioles. It indicated that transgelin may be an important potential target for the development of irreversible pulmonary vascular lesion in CHD patients.

Transgelin, a 22‐kD protein of the calponin family which is also known as SM22α,WS3‐10 or p27, is exclusively and abundantly expressed in the cytoskeleton of visceral and vascular smooth muscle cells of adult animals. It is also the earliest markers of smooth muscle differentiation. Studies have elucidated the regulating functions of transgelin, including actin cytoskeleton rearrangement, phenotypic modulation of vascular smooth muscle cells (SMC), SMC proliferation, cell migration and tumour suppression.^9^, ^10^, ^11^, ^12^ It is unknown how transgelin influence the PAH development in CHD. In immunohistochemical staining of the lung tissue, transgelin was obviously expressed in pulmonary arterial smooth muscle cells (PASMC) of the remodelled and thickened pulmonary arterioles, especially in irreversible CHD‐PAH group. We speculated that transgelin may influence the PASMC function and then promote the pulmonary vascular remodelling and irreversible CHD‐PAH occurrence.

Based on the research findings on lung tissue samples, further cellular experiment was carried out to illuminate the role of transgelin in regulating the function of PASMC. We demonstrated that knockdown and overexpression of transgelin can affect the PASMC phenotype, proliferation, apoptosis and migration.

## 2 METHODS

### 2.1 Patient enrolment and lung biopsy

According to multidisciplinary and translational approaches guideline,^13^ 14 CHD‐PAH patients diagnosed by right heart catheterization (RHC) who met the inclusion criteria and would undergo complete repair surgery were prospectively enrolled. Lung biopsy was performed during the repair surgery with patients’ informed consent. The control normal lung tissues were from patients who would undergo surgery for bronchial carcinoma (n = 6). The tissues were sufficiently distal from the tumour to ensure the normal phenotype of tissue cells. After a year's follow‐up, mean pulmonary arterial pressure (mPAP) were evaluated by RHC to determine the diagnosis of reversible (mPAP < 25 mm Hg, n = 10) and irreversible (mPAP ≥ 25 mm Hg, n = 4) PAH. Patients did not receive any drug treatment for PAH during the overall process.^8^ This study complied with the Declaration of Helsinki and was approved by the Institutional Review Board of Fuwai Hospital.

### 2.2 Differential proteomic analysis of the lung tissue in reversible, irreversible CHD‐PAH and control group

Protein supernatants of the lung tissue specimens (four from reversible group, four from irreversible group and six from control group) were extracted and reserved. iTRAQ reagent kit (Applied Biosystems,USA) were used for protein precipitation, digestion and iTRAQ‐labelling. The peptide mixture was separated with HPLC system (RIGOL 3220) and analysed by TripleTOFTM 5600 LC/MS/MS (Applied Biosystems Sciex). Protein identification and quantification for mass spectrometry was performed with the ProteinPilot software Beta (version 4.2, Applied Biosystems) in the Human UniProtKB/Swiss‐Prot database. The data was exported with PDST software system.

### 2.3 Qualitative and quantitative analysis of the expression of transgelin in the lung tissue of each group

In MS analysis, transgelin expression was significantly increased in the lung tissue of CHD‐PAH patients, and its expression level in irreversible group was 2.5‐fold than that of the reversible group.

To confirm the proteomic findings of transgelin, immunohistochemical staining and Western blot were performed for qualitative and quantitative analysis of the expression of transgelin in the lung tissue of CHD‐PAH patients and normal lung tissue.

### 2.4 Immunohistochemical staining

Neutral formalin‐fixed lung tissues were made into 4‐5 μm sections after dehydration and paraffin embedding. After heating for antigen retrieval, 3% H_2_O_2_ was used to block endogenous peroxidase. The sections were incubated with transgelin (SM22α) antibody (Abcam corporation) at 4°C overnight. After incubation of second antibody for 30 minutes at room temperature and DAB colouration, the slides were covered with resin, and then observed under microscope.

### 2.5 Western blot

The tissue of each group was digested with RIPA lysis buffer. Prepared protein specimens of each group were loaded to 4%‐12% PAGE electrophoresis precast gels (Invitrogen). IBlot^®^ transfer stacks that contained the required buffers and transfer membrane (nitrocellulose) were used for transfer with iBlot^®^ 2 dry blotting device (Invitrogen). After blocking by TBST with 5% non‐fat dry milk at room temperature for 2 hours, nitrocellulose membrane was incubated with antibody of transgelin at 4°C overnight. The NC membrane was washed with TBST three times followed by incubation with horseradish peroxidase‐conjugated anti‐rabit IgG for 2 hours. After a second wash with TBST, immunoblots were detected using a chemiluminescence kit (Thermo Scientific Piece, Waltham, MA, USA), and the radiographs were quantitated via densitometry (Science Imaging System, BioRad, Hercules, CA, USA).

### 2.6 Functional exploration of transgelin in human PASMC (hPASMC)

#### 2.6.1 Cell culture

Primary human PASMC (Sciencell, USA) was cultured 37°C and 5% CO_2_ with complete smooth muscle cell medium (SMCM) containing 2% foetal bovine serum, 2% smooth muscle cell growth supplement and penicillin/streptomycin solution (Sciencell).

#### 2.6.2 Lentiviral infection

Lentivirus/GV248‐siTAGLN (hU6‐MCS‐Ubiquitin‐EGFP‐IRES‐puromycin) and lentivirus/GV358 ‐TAGLN (Ubi‐MCS‐3FLAG‐SV40‐EGFP‐IRES‐puromycin) and their corresponding control lentiviruses, lentivirus/GV248 and lentivirus/GV358 respectively, were obtained from Genechem (Shanghai, China). The recombined lentiviral particles were diluted with complete SMCM. Polybrene was added to enhance the infection efficiency. About 3.5 × 10^4^‐4.5 × 10^4^ hPASMC per well were seeded in a six‐well plate. After cell culture for 24 hours, the medium was replaced by 0.5 ml of the infection solution (LV‐GV248, LV‐siTAGLN, LV‐GV358, LV‐TAGLN) per well. 12 hours later, the infection solution was replaced by complete SMCM. The hPASMC were harvested 84 hours after infection for cell morphological observation, mRNA and protein expression, cell proliferation, apoptosis and migration. The transduction efficiency was greater than 85% as measured by the green fluorescent protein.

### 2.7 Cell morphological observation by Coomassie Brilliant Blue staining

Cells were grown on coverslips in the six‐well plate. After the infection process, cells were washed twice with phosphate buffered saline (PBS). The cells were pre‐fixed with 2% paraformaldehyde for 10 seconds and washed once with PBS. After incubation with 1% Triton X‐100 for 30 minutes and thrice PBS washing, cells were fixed with 4% paraformaldehyde for 20 minutes and stained with Coomassie Brilliant Blue for 30 minutes. Following that, the coverslips were washed by distilled water three times, dried naturally, made transparent by xylene and sealed by neutral balsam. The coverslips were finally observed under microscope.

### 2.8 Quantitative real‐time polymerase chain reaction (RT‐PCR)

Total RNA from the cultured cells of each group was extracted using TRIZOL (Invitrogen, USA). Quantification was performed with a two‐step reaction process: reverse transcription (RT) and PCR. The RNA was reversely transcribed to cDNA using the PrimeScript™ RT Master Mix kit (TaKaRa, Japan). PCR was performed on the 7500 real‐time PCR system (Applied Biosystems, USA) using Power SYBR^®^ Green PCR Master Mix kit (Ambion, USA). The primers of target genes were designed in the laboratory and synthesized by TianyiBiotech, China. The sequences of the primers was as follows: transgelin, forward 5′‐3′(GGCTGGTGGAGTGGATCATAG), reverse 3′‐5′(CCAGCTTGCTCAGAATCACG); OPN, forward 5′‐3′(TCCAGTTGTCCCCACAGTAG), reverse 3′‐5′(CCATGTGTGAGGTGATGTCC); PCNA, forward 5′‐3′(ACTCGTCCCACGTCTCTTTG), reverse 3′‐5′(TTCATTGCCGGCGCATTTTA); α‐SMA, forward 5′‐3′(GCTGGCATCCATGAAACCAC), reverse 3′‐5′(GTACATAGTGGTGCCCCCTG); SM‐MHC, forward 5′‐3′(GAGCCAACATTGAGACCTAT), reverse 3′‐5′(CCTCCAAAAGCAAGTCACT); β‐actin, forward 5′‐3′(GTCATTCCAAATATGAGATGCGT), reverse 3′‐5′(GCTATCACCTCCCCTGTGTG). Each RT‐qPCR reaction mix consisted of 10 μl SYBR^®^ Green PCR Master Mix, 2 μl cDNA, 1 μl each primer and 6 μl nuclease‐free water, in a final volume of 20 μl. Thermal cycling conditions included an initial step at 95°C for 30 seconds, followed by 40 cycles at 95°C for 5 seconds and 60°C for 30 seconds. Each sample was run in triplicate for analysis. The expression levels of mRNAs were normalized to ACTB and were calculated using the 2^−ΔΔCt^ method.

### 2.9 Cell protein expression in Western blot

Proteins of the cultured cells of each group were extracted with RIPA lysis buffer. The process of electrophoresis, transfer membrane, antibody incubation and imaging was as described above. The anti‐SM22α (transgelin), anti‐osteopontin (OPN), anti‐alpha smooth muscle actin (α‐SMA), anti‐smooth muscle myosin heavy chain (SM‐MHC), anti‐Bax, anti‐Bcl‐2 antibodies were from Abcam, United Kingdom. The anti‐Cytochrome c, anti‐proliferating cell nuclear antigen (PCNA), anti‐Capase‐3 and β‐actin antibodies were from Cell Signaling Technology, United States. The primary antibody dilutions was prepared as follows: OPN‐1:5000, PCNA‐1:1000,α‐SMA‐1:1000, SM‐MHC‐1:1000, Bax‐1:5000, Bcl‐2‐1:500, Cyt‐c‐1:1000, Capase‐3‐1:1000, β‐actin‐1:1000.

### 2.10 Cell proliferation assay

Cell count and EdU staining assay were used to evaluate the cell proliferation after transfection. The cells were washed with PBS gentlely for two times to clear the residual medium. After detaching by 0.25% trypsin‐EDTA, the cells were resuspended in 1 ml DMEM. Number of the cells in each group was manually counted using a haemocytometer. Cell proliferation after treatment was also observed under microscope.

#### 2.10.1 EdU staining assay

Cells were cultured on the coverslips. Cell proliferation of the each group was determined using the Click‐iT^®^ EdU Alexa Fluor^®^ 647 Imaging Kit (Invitrogen, Thermo Fisher Scientific), according to the manufacturer's protocol. The cells of each group were first incubated with EdU working solution that had been diluted with cell medium at a concentration of 10 μM for 3 hours. After incubation, cells were fixed in 4% paraformaldehyde for 15 minutes at room temperature. Following two washes with 3% BSA in PBS, cells were permeated by 0.5% Triton^®^ X‐100 in PBS at room temperature for 20 minutes. Then the cells were incubated with the Click‐iT reaction cocktail (prepared according to the instructions) for 30 minutes, protected from light. Cell nuclei were stained with Hoechst 33342 at a concentration of 5 μg/mL for 30 minutes, protected from light. The coverslips were then washed and sealed by neutral balsam. Images were acquired using a fluorescence microscope (Leica Microsystem, Germany). The EdU‐positive (proliferative) cells were characterized with pink nuclei. Proliferation was presented as % proliferative cells. The assay was repeated for at least three times to achieve statistical analysis.

### 2.11 Cell apoptosis assay

Cell apoptosis was determined by terminal deoxyribonucleotidyl transferase mediated dUTP nick end labeling (TUNEL) assay and Annexin‐V flow cytometry.

#### 2.11.1 TUNEL assay

Cells were cultured on the coverslips. Apoptosis of the treated cells were detected using one‐step TUNEL cell apoptosis detection kit (red fluorescent protein labelling with TRITC) (KeyGEN BioTECH, China). The cells were fixed with 4% paraformaldehyde for 30 minutes, rinsed with PBS and permeabilized with 1% Triton X‐100 for 20 minutes. The cells then were incubated with TdT reaction mixture for 1 hour at 37°C, protected from the light. After washing with PBS, the cells were treated with streptavidin‐TRITC diluted with labelling buffer for 30 minutes at 37°C, protected from the light. Cell nuclei were stained with DAPI solution for 10 minutes at room temperature. The coverslips were then washed and sealed by neutral balsam. Images were acquired using a fluorescence microscope (Leica Microsystem, Germany). The TUNEL‐positive (apoptotic) cells were characterized with pink nuclei. Apoptosis was presented as normalize ratio of the percentage of the positive cells in experimental group and control group. The assay was repeated for at least three times to achieve statistical analysis.

#### 2.11.2 Annexin‐V flow cytometry

The cell apoptosis was detected using Annexin V/APC‐7ADD apoptosis detection kit (KeyGEN BioTECH, China) according to the manufacturer's instructions in the meanwhile. Treated cells were harvested and washed twice with ice‐cold phosphate buffer solution. Then the cells were resuspended in 500 ul binding buffer, incubated with 5 ul Annexin V‐APC and 5 ul 7‐amino‐actinomucin D (7‐ADD) for 15 minutes. All the samples were immediately analysed using a flow cytometer (Accuri C6, BD Biosciences, USA). The assay was repeated for at least three times to achieve statistical analysis.

### 2.12 Transwell migration assays

After transfection for 84 hours, cells were starved with DMEM (without FBS) for 12 hours. Following that, cells were digested and resuspended with DMEM. After cell counting with haemocytometer, 1 × 10^4^ of the resuspended cells in a volume of 200 ul were seeded in the transwell chamber located in a 24‐well plate. 1.1 ml of complete DMEM containing 20%FBS were added to the well. The cells were then cultured for 48 hours and then washed once with PBS. The un‐migrated cells on the upper chamber surface were wiped out with cotton swabs. The migrated cells were then fixed with 4% paraformaldehyde for 30 minutes, stained with crystal violet for 20 minutes and washed three times with PBS. Images were acquired using a fluorescence microscope (Leica Microsystem, Germany). Ten visual fields were randomly selected to calculate the average cells in each group. Result was presented as fold of the migrated cells. The assay was repeated for at least three times to achieve statistical analysis.

### 2.13 Statistical analysis

Statistical analysis was performed with spss 23.0 (IBM, USA). Data are presented as a mean ± standard deviation (SD). The correlation of expression of transgelin and pathological grading was analysed by Spearman's correlation. Comparisons among groups were assessed with Student's *t* test or ANOVAs. *P* value less than 0.05 was considered statistically significant.

## 3 RESULTS

### 3.1 Transgelin was significantly up‐regulated in the pulmonary arteria of irreversible CHD‐PAH

**Figure 1:**
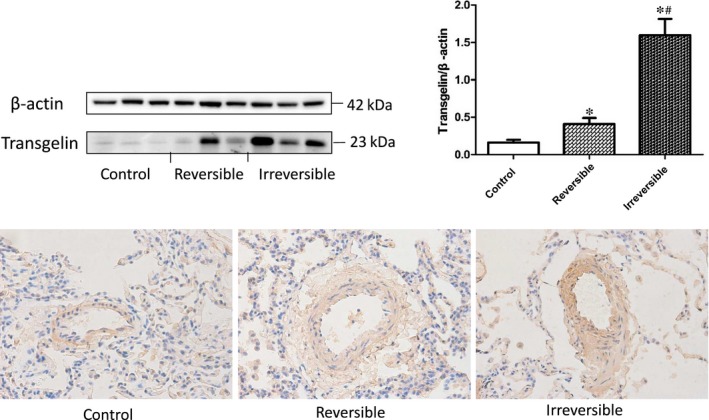
Transgelin expression in the lung tissue of reversible, irreversible and control group in immunohistochemical staining (40X) and Western blot test. The result data were represented as X ± SD. *: reversible or irreversible CHD‐PAH group vs control group, *P* < 0.05. #: reversible vs irreversible CHD‐PAH group, *P* < 0.05

As in previous proteomic analysis, transgelin was significantly up‐regulated in the irreversible CHD‐PAH group. In qualitative and location analysis, immunohistochemical staining and Western blot test confirmed the same findings. Transgelin was expressed obviously in the PASMC of the middle pulmonary arterioles, especially in the irreversible PAH group in immunohistochemical staining. Western blot also showed an uptrend from control group to reversible group and irreversible group, and the differences between groups were significant. (Figure 1) In correlation analysis, transgelin showed to be positively related with pathological grading (*R* = 0.95, *P* < 0.001). These indicated that transgelin may be an important factor in the development of CHD‐PAH.

### 3.2 Transgelin induces morphological changes in hPASMC

**Figure 2:**
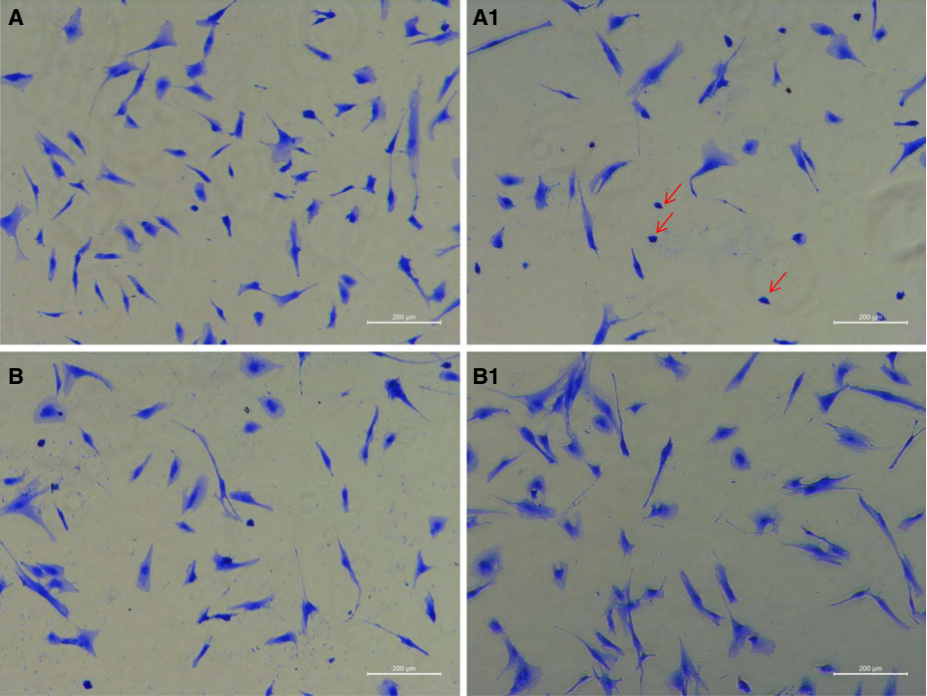
HPASMC morphology change in Coomassie Brilliant Blue (CBB) staining(100X). A0: LV‐GV248, A1: LV‐siTAGLN; B0: LV‐GV358, B1: LV‐TAGLN. The red arrows showed the small, round and punctiform hPASMC in LV‐siTAGLN group

Coomassie Brilliant Blue (CBB) staining showed obvious morphological changes in LV‐siTAGLN and LV‐TAGLN group. (Figure 2) The normally grown primary hPASMC exhibited to be polymorphic, most of them were spindle‐shaped, while some were irregular. In LV‐siTAGLN group, many cells turned to be small, round and punctiform (as the red arrows showed), which were similar to the apoptotic cells with cell shrinkage. And the cells were much sparser than in the control group. While in LV‐TAGLN group, cells did not show much difference in shape but the cells were much thicker and stronger than the control cells. The morphological staining may gave us an initial impression that reducing TAGLN expression can lead to hPASMC apoptosis, while TAGLN overexpression strengthened the cytoskeleton.

### 3.3 Effect of transgelin on the expression of phenotype markers of hPASMC

**Figure 3:**
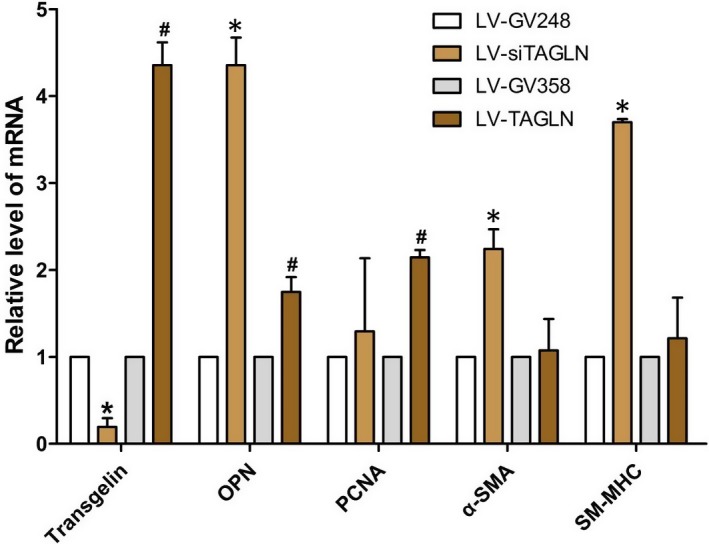
The expressions levels of mRNAs of phenotypic markers of hPASMC in real‐time PCR. The resultant data were represented as X ± SD. *: LV‐siTAGLN vs LV‐GV248, *P* < 0.05. #: LV‐TAGLN vs LV‐GV358, *P* < 0.05

**Figure 4:**
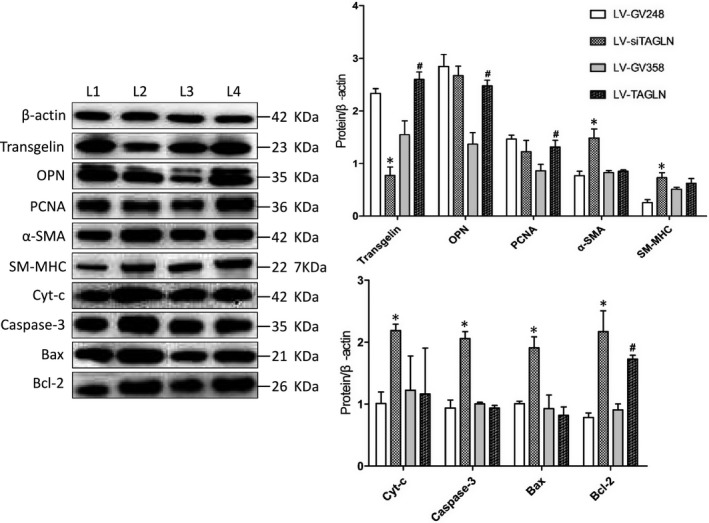
The expression of phenotype markers of hPASMC and apoptosis‐related proteins in Western blot test. L1: LV‐GV248, L2: LV‐siTAGLN, L3: LV‐GV358, L4: LV‐TAGLN. The resultant data were represented as X ± SD. *: LV‐siTAGLN vs LV‐GV248, *P* < 0.05. #: LV‐TAGLN vs LV‐GV358, *P* < 0.05

The markers of expression of contractile phenotype (α‐SMA, SM‐MHC) and synthetic phenotype (OPN, PCNA) of smooth muscle cells were detected in real‐ timePCR and Western blot (WB) test. (Figures 3 and 4) It showed thatα‐SMA, SM‐MHC expression were significantly increased in the both mRNA and protein level in LV‐siTAGLN group, while in LV‐TAGLN group, it was no different than the control group. In WB test, OPN and PCNA protein expression were increased in cells of LV‐TAGLN group, but protein levels were found to be similar in LV‐siTAGLN and control group. Real‐time PCR exhibited the same findings with WB for PCNA. But for OPN, the mRNA level was increased in both LV‐siTAGLN and LV‐TAGLN group, especially in the LV‐siTAGLN group. The results indicated that transgelin can influence the expression of phenotype markers of PASMC, suppressing the expression of transgelin that promoted the contractile phenotype markers (α‐SMA, SM‐MHC) expression, while transgelin overexpression induced the expression of synthetic phenotype markers (OPN, PCNA). And the influence of transgelin on OPN differed in mRNA and protein expression. The mRNA level of OPN presented to be significantly up‐regulated as transgelin expression decreased, while the protein did not show much difference within the control cells. We may speculate that the mRNA translation process of OPN was closely related with the expression level of transgelin and the depression in transgelin would significantly influence the protein expression of OPN. However, the molecular mechanism will need further exploration.

### 3.4 Transgelin influences proliferation of hPASMC

**Figure 5:**
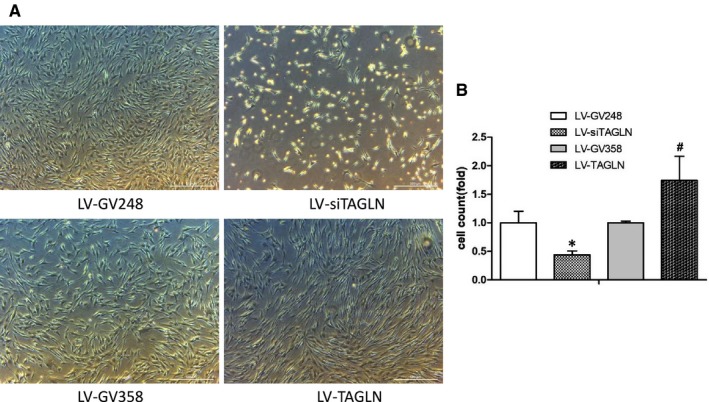
HPASMC proliferation in cell counts. A, cell proliferation observed under microscope (50X) after treatment. B, cell counts with haemocytometer.*: LV‐siTAGLN vs LV‐GV248, *P* < 0.05. #: LV‐TAGLN vs LV‐GV358, *P* < 0.05

**Figure 6:**
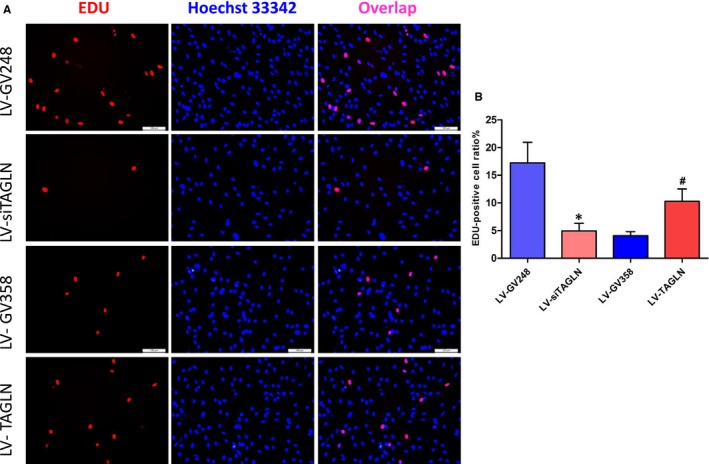
HPASMC proliferation in EdU‐647 cell proliferation assay. The EdU‐positive (proliferative) cells were characterized with pink nuclei. The resultant data were represented as X ± SD. *: LV‐siTAGLN vs LVGV248, *P* < 0.05 #: LV‐TAGLN vs LV‐GV358, *P* < 0.05

Cell count analysis presented that the number of living cells was much less in LV‐siTAGLN group while significantly more in LV‐TAGLN group (*P* < 0.05 for both). That accorded with the cell growth status that observed under microscope. The cells were obviously sparser after the suppression of transgelin expression (LV‐siTAGLN), while cells with transgelin overexpression presented a higher cellular density (LV‐TAGLN). (Figure 5) EdU‐647 cell proliferation assay showed that hPASMC proliferation was much lower in LV‐siTAGLN group and higher in LV‐TAGLN group (*P* < 0.05) (Figure 6). This may be in line with OPN and PCNA expression in WB, which can reflect the cell proliferation indirectly. Transgelin overexpression enhanced hPASMC proliferation while suppressing the expression of transgelin‐decreased cell proliferation.

### 3.5 Transgelin influence apoptosis of hPASMC

**Figure 7:**
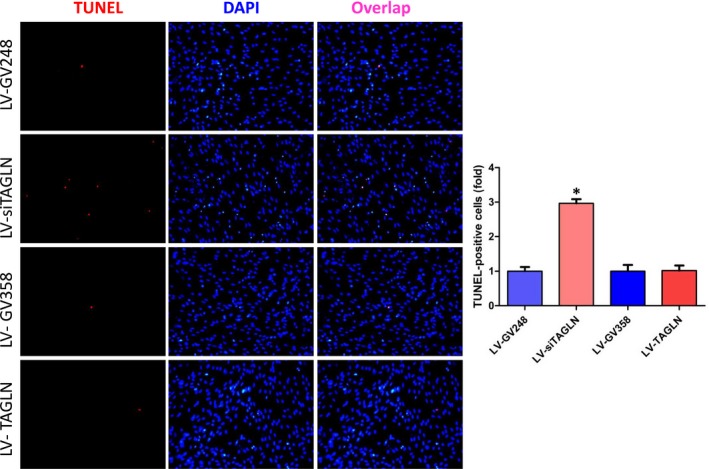
HPASMC apoptosis in one‐step TUNEL cell apoptosis detection. The TUNEL‐positive (apoptotic) cells were characterized with pink nuclei. The resultant data were represented as X ± SD. *: LV‐siTAGLN vs LV‐GV248, *P* < 0.05

**Figure 8:**
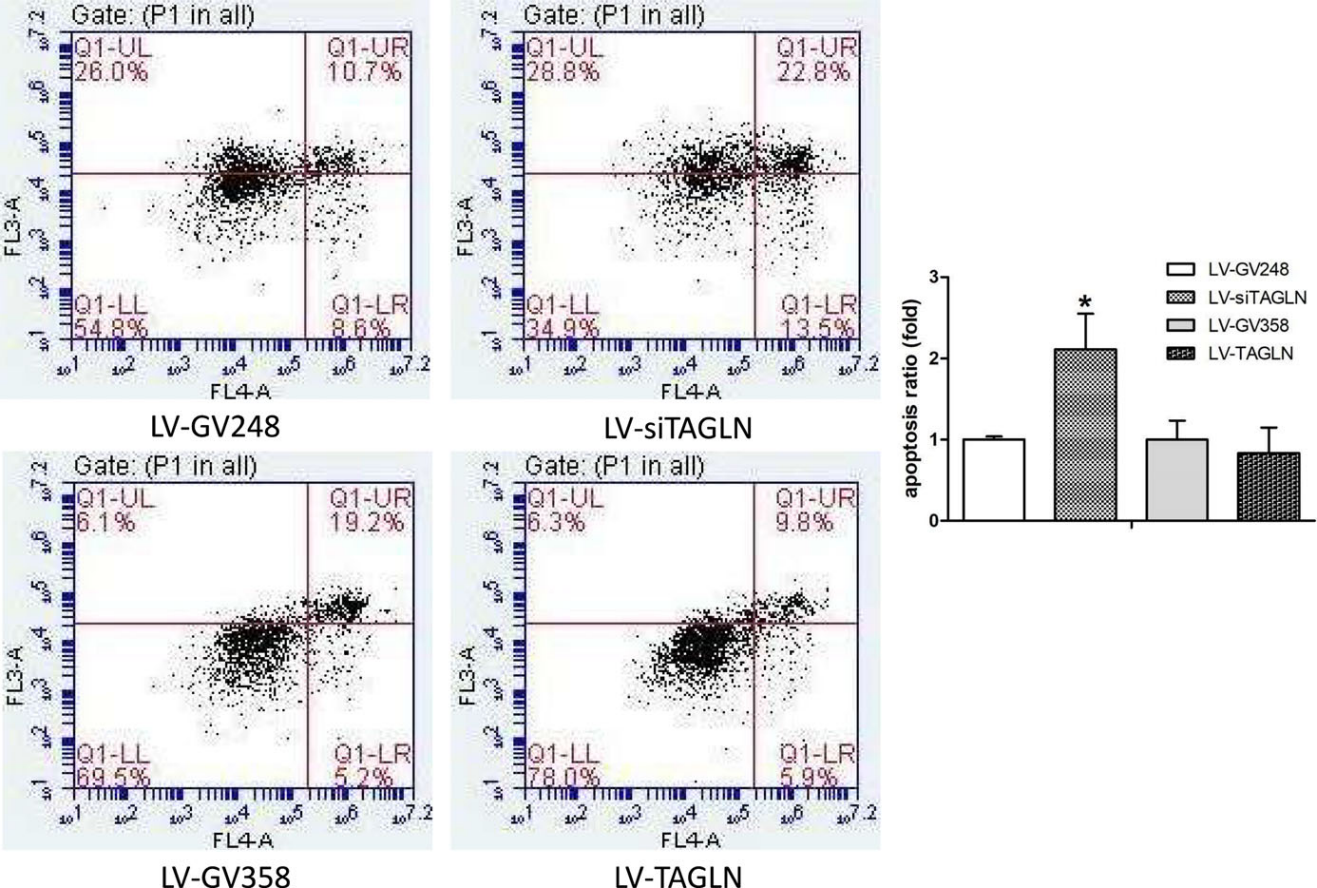
HPASMC apoptosis in Annexin‐V flow cytometry. The apoptosis ratio as calculated with the early‐ and late‐stage apoptotic cells in the right quadrant. *: LV‐siTAGLN vs LV‐GV248, *P* < 0.05. #: LV‐TAGLN vs LV‐GV358, *P* < 0.05

The results of TUNEL assay (Figure 7) and Annexin‐V flow cytometry (Figure 8) showed a significant increase in hPASMC apoptosis ratio in LV‐siTAGLN group (*P* < 0.05), while the apoptosis ratio was similar in LV‐TAGLN group and its control group. In WB (Figure 3B), the apoptosis‐related proteins (cytochrome c, caspase 3, bax, bcl‐2) were significantly up‐regulated in LV‐siTAGLN group, which may indicate an active apoptosis process in the LV‐siTAGLN cells. While in LV‐TAGLN group cytochrome c, caspase 3 and bax expression were not different than those in the control group, except that the anti‐apoptotic protein bcl‐2 was obviously expressed in LV‐TAGLN group, which may indicate an anti‐apoptotic character in the LV‐TAGLN cells. These results may reveal that suppressing the expression of transgelin can induce hPASMC apoptosis and that transgelin overexpression endowed hPASMC with resistance to apoptosis.

### 3.6 Transgelin influenced migration of hPASMC

**Figure 9:**
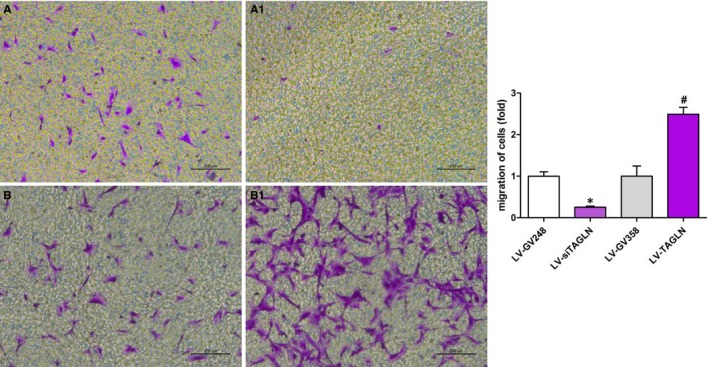
HPASMC migration ratio in transwell migration assays. A0: LV‐GV248, A1: LV‐siTAGLN; B0: LVGV358, B1: LV‐TAGLN. The resultant data were represented as X ± SD. *: LV‐siTAGLN vs LV‐GV248, *P* < 0.05 #: LV‐TAGLN vs LV‐GV358, *P* < 0.05

Transwell migration assays showed a distinct effect of transgelin on the migration of hPASMC, as it shown in Figure 9. HPASMC migration in LV‐TAGLN cells was significantly increased as compared with the control group and in LV‐siTAGLN cells it was significantly decreased. We may conclude that transgelin overexpression induced hPASMC migration, while suppressing the expression of transgelin restrained the migration of the cells.

## 4 DISCUSSION

In this study, we first demonstrated that transgelin was significantly up‐regulated in the lung tissue of CHD‐PAH patients and maybe closely related to the development of irreversible pulmonary vascular pathologic changes. PASMC phenotype, proliferation, apoptosis and migration were significantly affected by transgelin. Transgelin overexpression induced hPASMC phenotype change, proliferation and migration, while knockdown of transgelin led to cell apoptosis and decreased cell proliferation and migration.

### 4.1 Transgelin and CHD‐PAH development

Studies indicated that the severity of pulmonary vasculature remodelling adversely influenced the natural history and operative mortality in CHD‐PAH patients.^14^, ^15^, ^16^ But the mechanism of pulmonary remodelling is poorly understood in irreversible CHD‐PAH. Transgelin as an important cytoskeletal protein has been demonstrated to be present with several biological functions in SMC. Previous studies had shown a possible role of transgelin in hypoxic pulmonary hypertension (HPH) and pulmonary vascular remodelling under hypoxia.^12^, ^17^ In our explorative and comparative research of the lung tissue from reversible and irreversible CHD‐PAH patients and normal lung tissue, transgelin was found to be preferentially expressed in the remodelled pulmonary arterioles of CHD‐PAH patients, especially in PASMC of the pulmonary artery media from irreversible CHD‐PAH patients. And the expression level of transgelin was consistent with the pathological degree of the pulmonary arteries of reversible and irreversible PAH lung tissues. It reminds us that transgelin is closely related to the development of CHD‐PAH and maybe an important target in the irreversibility of CHD‐PAH, while transgelin expression and PASMC proliferation may play a role in the pathological process. In the development of PAH, abnormal proliferation, migration and apoptosis of PASMC‐induced pulmonary vascular remodelling constitutes the main reason. Studies showed that transgelin expression was increased in hPAMSCs under hypoxia accompanied with increased cell proliferation and migration, while inhibition of the expression of transgelin impaired the PASMC proliferation and migration under hypoxia.^12^, ^17^ Nevertheless, it was indeterminate whether the proliferation and migration changes in hPAMSCs under hypoxia were the direct biological effects of increased transgelin expression or the indirect effects of other factors that were stimulated by hypoxia. As it is known, the mechanism of pulmonary vascular remodelling of CHD‐PAH is quite different from that of hypoxic pulmonary hypertension (HPH). It will be an important aspect to figure out the function of transgelin on PASMC.

### 4.2 Transgelin and PASMC proliferation, migration, apoptosis and phenotype

In studies, transgelin was demonstrated with a divergent function on cell proliferation, migration and apoptosis under different conditions. Daniel's study demonstrated that transgelin was up‐regulated in repopulating mesangial cells and promoted their migratory and proliferative nature after injury.^18^ It was reported in another study that increased alveolar epithelial type II cell expression of transgelin contributed to cell migration in lung fibrosis through TGF‐β/Smad3 pathway.^19^ In contrast, study also found that overexpression of transgelin inhibited VSMC proliferation and neointima formation via blockade of the Ras‐extracellular signal‐regulated kinase (ERK) 1/2 pathway.^20^ Transgelin even presented different regulating effects of migration on VSMC under different cell phenotype.^21^ Transgelin which was considered as a tumour suppressor was shown to suppress proliferation of HepG2 cells, while the transgelin ‐overexpressing cells became resistant to apoptotic cell death caused by cytotoxic agents.^22^

In the present study, transgelin overexpression activated PASMC proliferation and migration distinctly, and meanwhile the PASMC was likely anti‐apoptotic as the anti‐apoptotic protein (bcl‐2) was significantly increased. Inhibition of transgelin expression presented to trigger cell apoptosis in PASMC, while depressing the cell proliferation and migration. The obviously expressed apoptosis‐related protein (cyt‐c, caspase 3, bax) indicated an active mitochondrial apoptotic pathway in these cells. Despite the increasing knowledge on the function of transgelin, the various molecular mechanisms of PASMC proliferation, migration and apoptosis influenced by transgelin include mediating signalling pathways in PAH that remain largely elusive and needed further exploration.

Studies demonstrate that VSMCs have two phenotypes. In normal and mature blood vessel wall, VCMCs are mainly exhibited in contractile phenotype. VSMC can turn to synthetic phenotype during abnormal microenvironments causing endothelial lesions and cytokines release, which is companied by cell proliferation, migration, synthesis of extracellular matrix, increased expression of synthetic markers (PCNA, OPN) and decreased expression of contractile marker molecules (α‐SMA, SM‐MHC).^23^, ^24^ VSMC phenotype switch was involved many diseases, such as atherosclerosis, restenosis and hypertension.^25^ Phenotype change‐induced PASMC proliferation and vascular remodelling also play an important role in HPH.^26^, ^27^, ^28^ Meanwhile, transgelin was suggested to maintain the contractile phenotype of SMCs and inhibits the phenotypic modulation of SMCs from contractile to synthetic/proliferative cells during atherosclerosis.^29^ However, the molecular mechanism of phenotypic transition presented unknown, the reorganization of the actin cytoskeleton seemed to be involved in that process.^30^, ^31^, ^32^

In this in vitro experiment, transgelin seemed to regulate the PASMC phenotype in a quite different way. The overexpression of transgelin promoted the expression of synthetic markers (OPN, PCNA) and induced cell proliferation and migration. However, the cell morphology did not show obvious changes, except that the cells seemed to obtain strengthened cytoskeleton. It was reported that SM22α knockout resulted in increased atherosclerotic lesion area and a higher proportion of proliferating SMC‐derived plaque cells in animal models of atherosclerosis.^29^ Studies also demonstrated a decrease in transgelin expression in the synthetic VSMC induced by cytokines like TGF‐β.^23^ But in this study, inhibition of transgelin expression directly did not turn the hPASMC into synthetic phenotype but mainly promoted cell apoptosis. We may think the VSMC phenotype change that reported in the atherosclerosis animal model is not the direct regulation of transgelin on VSMC but the role of the cytokines that holding true.

### 4.3 The possible pathways that are involved in transgelin and PASMC function in CHD‐PAH

TGF‐β/smad signalling, as a canonical upstream regulating pathway of transgelin, was demonstrated to involve in hypoxia‐induced PH, pulmonary vascular remodelling, PASMC proliferation and migration. Protein interaction pathway analysis of the differential expression proteins in our previous work showed a possible activation of TGF‐β/smad pathway, which indicated that TGF‐β/smad pathway might be one of the possible regulatory mechanisms that is responsible for transgelin changes in CHD‐PAH.

Previous studies have demonstrated various regulating functions of STAT3 signalling in PAH development.^33^, ^34^ STAT3 activation was found in both human and experimental models of PAH.^35^ Haemoodynamic forces which were induced by pressure overload, shear stress and cyclic strain were also upstream activators of STAT3.^34^ That may remind us the possible activation of STAT3 induced by the high haemodynamics in CHD‐PAH. Studies also revealed the regulating role of STAT3/NFAT/Ca^2+^‐K^+^channels in PAH development.^34^ NFAT (nuclear factor of activated T cells) and Ca(2+)/calcineurin‐sensitive transcription factor, were shown to down‐regulate the K+ channels, leading to cell depolarization, increased calcium levels and cell proliferation. NFATc2 activation up‐regulated bcl‐2 leading to apoptosis resistance.^36^, ^37^ Flow shear stress was reported to enhance intracellular Ca^2+^ signalling in PASMC from patients with PAH.^38^ Transgelin, as an important marker of PASMC, was closely related to pulmonary vasoconstriction and pulmonary arterial medial hypertrophy, as well as Ca^2+^signalling. Thus, we might predict some interactions between high haemodynamics (flow shear stress), STAT3/NFAT, transgelin and Ca^2+^signalling in CHD‐PAH as well.

Study have reported an up‐regulation of RAGE in pulmonary arterioles of PAH patients, as well as experimental PAH models. Receptor for advanced glycation end‐products (RAGE) activation promoted PASMC proliferation and resistance to apoptosis. This raised doubts on the important role of RAGE on PAH development.^39^ Studies in vitro reported that RAGE activation decreased the expression of transgelin on both mRNA and protein levels, and interfered with the contractile phenotype of VSMC,^40^ and RAGE activation‐induced apoptosis in VSMC.^41^ We may also consider RAGE as an important upstream regulating factor of transgelin in CHD‐PAH although the increased RAGE repression did not reach a significant level in our previous proteomics analysis. However, the expression of transgelin in our study of CHD‐PAH was significantly up‐regulated, and the increased transgelin promoted PASMC proliferation and resistance to apoptosis. The regulatory mechanism of RAGE and transgelin seemed to be complicated and we suspect that some other signals may also be involved in.

Proteomics analysis of our previous work also showed obvious activation of adhesion‐integrin‐mediated cell adhesion and migration. FAK, which is activated by growth factor receptors and integrin clustering is required for cell adhesion, migration and other aspects of the remodelling process. FAK activation was closely related with VSMC proliferation, migration and phenotypes.^42^, ^43^ FRNK, a specific FAK inhibitor, was selectively expressed in large arterioles when SMC are transitioning from a synthetic to contractile phenotype, and inhibited FAK‐dependent SMC proliferation and migration. FAK activation significantly reduced SM22 (transgelin) and α‐SMA expression,^42^ leading to cell proliferation and modulation from a contractile phenotype to a synthetic phenotype.^44^ Studies also showed that FAK activation requires signalling by both the growth factor receptors and the fibronectin‐binding integrin.^44^ TGF‐β could induce FRNK expression and then restrained FAK activation, which finally stimulated SM marker gene expression.^42^ These results may remind us that FAK is an upstream regulating factor of transgelin, and the abnormal expression level of growth factor and integrin under PAH pathologic status can influence PASMC proliferation, migration and phenotypes through FAK. And that progress maybe somewhat involved with TGF‐β activation. TGF‐β/FAK/transgelin may be another possible pathway in the development of CHD‐PAH.

Thus, we speculated that transgelin may promote pulmonary vascular remodelling in PAH through influencing cell phenotype, proliferation, migration and apoptosis of PASMC. Transgelin overexpression in PASMC in pulmonary arterioles may activate cell proliferation, migration and inhibit cell apoptosis, which results in pulmonary vascular remodelling and finally lead to irreversible pulmonary vasculopathy in CHD‐PAH. TGFβ/smad, STAT3/NFAT/Ca2+, RAGE and TGF‐β/FAK may be possible pathways which regulate transgelin expression and PASMC function and participate in CHD‐PAH development. Further exploration under CHD‐PAH pathological conditions (fluid shear stress and CHD‐PAH rat model) will be needed to better illustrate the role of transgelin in CHD‐PAH.

## 5 LIMITATION

We also have some limitations in this study. First, we lack the animal experimental research to test and verify the role of transgelin in the development of CHD‐PAH and irreversible pulmonary vasculopathy. Second, we failed to explore the upstream mechanism of the increased expression of transgelin in the pulmonary arterioles of CHD‐PAH patients under the present research conditions on lung tissue and in vitro cell lines. Our future research work will explore these discrepancies.

## 6 CONCLUSIONS

We conclude that transgelin is a potential important target in the development of irreversible CHD‐PAH. The up‐regulated transgelin‐induced phenotype change, proliferation, migration and anti‐apoptosis of PASMC may promote pulmonary arterioles remodelling, which may in turn finally lead to irreversible pulmonary vasculopathy in CHD‐PAH. Further in vivo and in vitro research on transgelin and CHD‐PAH development and their corresponding pathways and mechanisms are needed.

## CONFLICT OF INTEREST

The authors declare no relevant financial involvement with any organization or entity with a financial interest in or financial conflict with the subject matter or materials discussed in the manuscript.
